# The *P450* multigene family of *Fontainea* and insights into diterpenoid synthesis

**DOI:** 10.1186/s12870-021-02958-y

**Published:** 2021-04-20

**Authors:** Shahida A. Mitu, Steven M. Ogbourne, Anne H. Klein, Trong D. Tran, Paul W. Reddell, Scott F. Cummins

**Affiliations:** 1 GeneCology Research Centre, University of the Sunshine Coast, Maroochydore DC, Queensland 4558 Australia; 2 School of Science, Technology and Engineering, University of the Sunshine Coast, Maroochydore DC, Queensland 4558 Australia; 3 EcoBiotics Ltd, Yungaburra, Queensland 4884 Australia

**Keywords:** Anti-cancer, EBC-46, Tigilanol tiglate, Transcriptome-wide identification, Biosynthesis

## Abstract

**Background:**

Cytochrome P450s (P450s) are enzymes that play critical roles in the biosynthesis of physiologically important compounds across all organisms. Although they have been characterised in a large number of plant species, no information relating to these enzymes are available from the genus *Fontainea* (family Euphorbiaceae). *Fontainea* is significant as the genus includes species that produce medicinally significant epoxy-tigliane natural products, one of which has been approved as an anti-cancer therapeutic.

**Results:**

A comparative species leaf metabolome analysis showed that *Fontainea* species possess a chemical profile different from various other plant species. The diversity and expression profiles of *Fontainea* P450s were investigated from leaf and root tissue. A total of 103 and 123 full-length *P450* genes in *Fontainea picrosperma* and *Fontainea venosa*, respectively (and a further 127/125 partial-length) that were phylogenetically classified into clans, families and subfamilies. The majority of *P450* identified are most active within root tissue (66.2% *F. picrosperma,* 65.0% *F. venosa*). Representatives within the CYP71D and CYP726A were identified in *Fontainea* that are excellent candidates for diterpenoid synthesis, of which CYP726A1, CYP726A2 and CYP71D1 appear to be exclusive to *Fontainea* species and were significantly more highly expressed in root tissue compared to leaf tissue.

**Conclusion:**

This study presents a comprehensive overview of the *P450* gene family in *Fontainea* that may provide important insights into the biosynthesis of the medicinally significant epoxy-tigliane diterpenes found within the genus.

**Supplementary Information:**

The online version contains supplementary material available at 10.1186/s12870-021-02958-y.

## Background

Diterpenes, also known as diterpenoids or isoprenoids, are a structurally diverse class of small molecules that are widespread throughout the plant kingdom. Diterpenes exhibit many and varied biological activities and consequently there is significant commercial interest in their potential applications as pharmaceuticals, food products, and industrial and agricultural chemicals [1–5]. Tigilanol tiglate (TT), a novel epoxy-tigliane diterpene ester extracted from the fruit of *Fontainea picrosperma* (family Euphorbiaceae) [3, 6], is of particular current interest due to its effectiveness as a local treatment for a range of cancers in humans and companion animals [3, 7–9]. Recently, TT was approved by regulatory authorities in Europe and the USA as a veterinary pharmaceutical for the treatment of non-metastatic canine mast cell tumours.

TT cannot be synthesised on a commercial scale and instead is obtained by purification from the fruit of *F. picrosperma* [10]. Despite this, all tissues of plants may accumulate diterpenoids [11], so whilst the fruit of *F. picrosperma* is the raw material for the purification of TT, TT is likely present in all tissues of the plant. To this point, the natural biosynthetic pathway leading to the biosynthesis of tigliane esters such as TT is currently unknown, but as is the case for other macrocyclic diterpenes (e.g. jatrophane & ingenane), the roots may be the site of biosynthesis [12, 13]. Given the important role cytochrome P450s (P450s) play in the biosynthetic pathways of both primary and secondary metabolite production [14], it is likely that they are critical to the biosynthesis of TT and epoxy-tiglianes more generally.

P450s are widely distributed in eukaryotes, where they form a large and diverse class of enzymes consisting of more than 35,000 members [15, 16] and play vital roles in biosynthesis of natural products, degradation of xenobiotics, biosynthesis of steroid hormones, drug metabolism and synthesis of secondary metabolites [14, 15]. They catalyse reactions in biosynthetic pathways of many compounds such as alkaloids, flavonoids, lignans, isoprenoids, phenolics, antioxidants and phenylpropanoid [17, 18]. In plants, the P450 superfamily is one of the largest gene families of enzyme proteins, where for instance, it is the third largest gene family present in *Arabidopsis* [19]. At present, 5100 plant P450s have been annotated and clustered into two different categories (A type and non-A-Type) and 11 different clans [20]. The A-type P450 enzymes are grouped as the CYP71 clan, whereas the non-A type are subdivided into 10 clans - CYP51, CYP72, CYP74, CYP85, CYP86, CYP97, CYP710, CYP711, CYP727, and CYP746 [14, 15, 19] according to the standard nomenclature system [21]. The CYP71 clan includes more than 50% of all plant P450s [22, 23].

Despite the abundance of diterpenoids in plants, there are still large gaps in our understanding of their biosynthesis pathways. To date, CYP71D and CYP726A members (within clan CYP71) have been functionally characterized as diterpene modifying in Euphorbiaceae [24, 25]. Furthermore, these P450 genes are present in genome biosynthetic clusters, also with casbene synthase, and this organisation is conserved across the Euphorbiacae species investigated (i.e. for those species from which genome is available) [26–29]. This has led to the theory that P450s are the driving force for plant diterpene diversity [26]. Based on only a small pool of experimental gene expression data, these diterpenoid biosynthetic genes have broad tissue expression, from leaf to root, flower and stem [25, 30].

To date, there are no reports of *P450* genes in *Fontainea*, yet the next-generation sequencing (NGS) approach provides the ideal tool towards their elucidation in this genus. *Fontainea picrosperma* and *Fontainea venosa* are closely related species that presumably produce similar arrays of natural products, including epoxy-tigliane diterpenes. In this study, we report the general metabolomic profiles of these two *Fontainea* species, and compared to non-*Fontainea* species. Towards better understanding these similarities and differences, we used NGS trancriptomics to elucidate the *Fontainea P450* family and their relative gene expression in leaf and root tissue, with particular focus on those predicted to be involved in diterpenoid synthesis.

## Results

### Metabolomic analysis and P450 identification

**Fig. 1:**
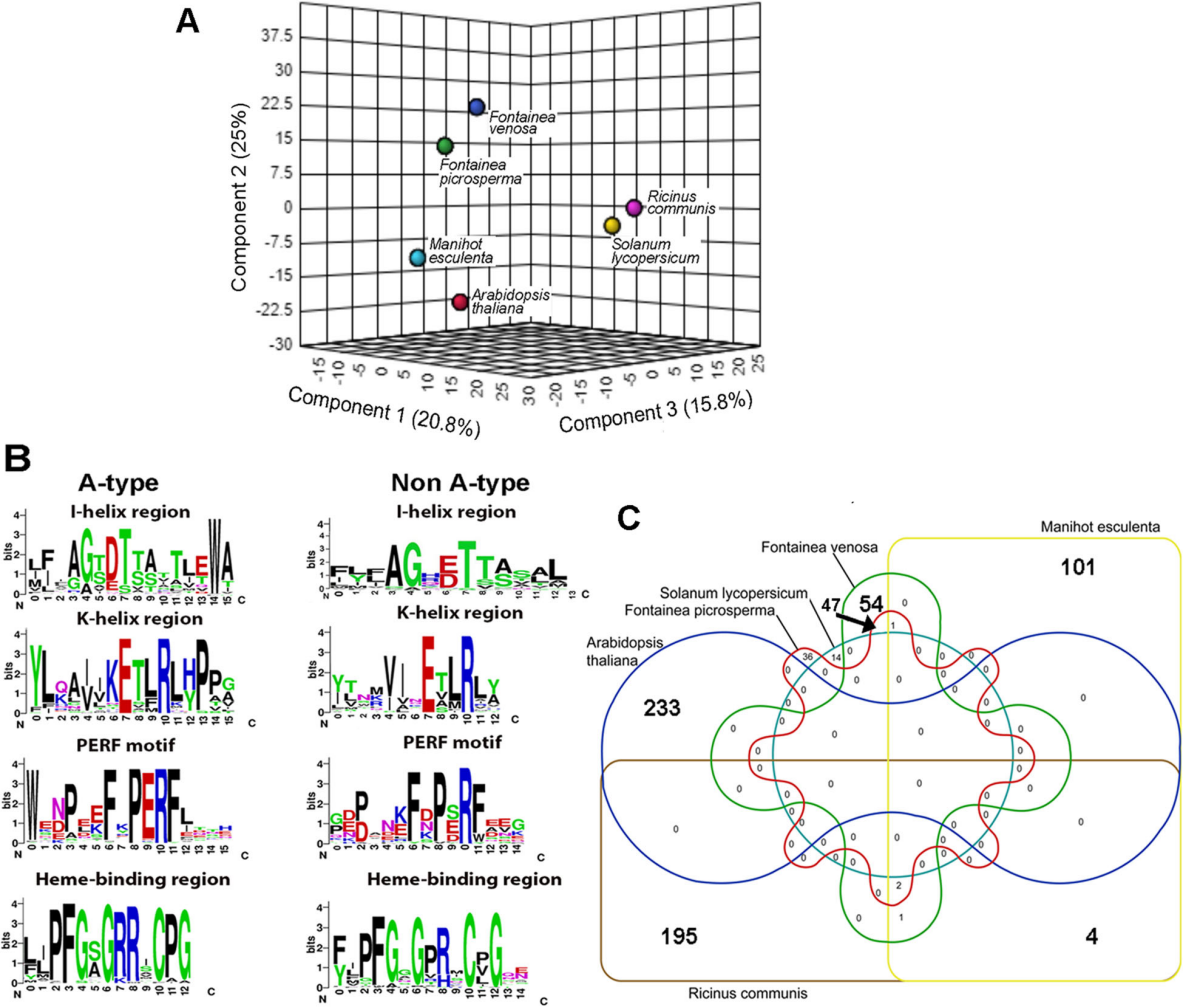
Comparative metabolome and P450 analysis in *Fontainea* and 4 other non-*Fontainea* plant species. **a** Partial least square-discriminant analysis (PLS-DA) derived from leaf tissue of six different plants according to their metabolome. **b** A-type and non A-type conserved motifs in *Fontainea* species P450 represented by weblogo. Motif names are shown above each logo. **c** Venn diagram showing P450 homology matches (> 90% identity) across the 6 plant species after pairwise comparison

Targeted analysis for TT in various tissues (root, leaf, bark and fruit) of *F. picrosperma* demonstrated its presence in all tissues (Additional Fig. 1). Based on this result, as well as prior evidence that diterpenoid biosynthesis enzymes are broadly expressed [31, 32], the leaf was chosen for a multi-species metabolomics comparison. Metabolomics analysis of leaf tissue from *F. picrosperma, F. venosa* and 4 other non-*Fontainea* plant species provided a total of 49,098 mass spectral ions extracted from the LC-MS dataset. A partial least square-discriminant analysis (PLS-DA) was performed to analyse the chemodiversity among samples (DOI: 10.25907/00049). The PLS-DA model with three components accounting for 20.8, 25.0 and 15.8% of the total variance showed that *F. picrosperma* and *F. venosa* were notably separated from other species (Fig. 1a). This untargeted metabolomic analysis indicated that *Fontainea* species were considerably more closely related from a chemical perspective compared to two Euphorbiaceae (*Manihot esculenta, Ricinus communis*) and two non-Euphorbiaceae (*Arabidopsis thaliana, Solanum lycopersicum*) plants.

To investigate if chemodiversity is correlated with P450 diversity, the *F. picrosperm*a and *F. venosa* P450 were initially identified through NGS, de novo reference assembly and gene ontology. Transcriptome libraries were constructed from combined root and leaf tissue for both *F. picrosperm*a and *F. venosa* plants using Illumina HiSeq 2500 trimmed reads (150–200 bp). In total, 12 Gb and 30 Gb raw reads were generated for *F. picrosperma* and *F. venosa* libraries, respectively, which were assembled into 192,639 (N50 1450 bp) and 246,608 (N50 1248 bp) contigs. From these reference *Fontainea* transcriptomes, we identified 103 and 123 full-length *P450* genes (and 127 and 125 partial-length) in *F. picrosperma* and *F. venosa,* respectively, all of which contained the conserved cytochrome P450 domain. The 4 different recognised P450 motif/regions (I-helix, K-helix, PERF and heme-binding [33]) are presented with noted conservation (Fig. 1b). A comparative P450 sequence identity analysis of the same 6 species used in metabolomic analysis, based on 90% sequence identity, demonstrated that *Fontainea* share considerably more P450 identity (48%) compared to other species of Euphorbiaceae and non- Euphorbiaceae (Fig. 1c)*.* The non- Euphorbiaceae species of *A. thaliana* and *S. lycopersicum* shared no P450 (> 90% identity) with *F. picrosperma* or *F. venosa*.

*F. picrosperma P450* genes were classified into 10 clans, within which there were 37 families and 45 subfamilies (Table 1**)**. In *F. venosa, P450* genes were classified into 9 clans, containing 37 families and 67 subfamilies (Table 2). The general characteristics of *Fontainea* full-length P450 proteins were also investigated, including the amino acid (aa) length, molecular weight, isoelectric point (pI) and presence of secretory signal peptide. Length varied from 300 to 618 aa in *F. picrosperma* and 301–632 aa in *F. venosa*, with molecular weights ranging from ~ 27–71 kDa. The pI values ranged from 5.22–9.8 in *F. picrosperma* and 5.29–9.91 in *F. venosa*. We also calculated their instability index (II) and found that 18 *F. picrosperma* and 52 *F. venosa* P450 were stable (stability factor < 40). The GRAVY values were negative for all P450 proteins, indicating them to be hydrophilic. In *F. picrosperma*, 68 P450 were predicted to have a secondary pathway signal peptide, whereas only 5 sequences (FpCYP74B2, FpCYP707A2, FpCYP707A3, FpCYP707A4 and FpCYP88A1) contained chloroplast-targeting peptides. In *F. venosa,* 78 P450 had pathway signal peptides and 3 (FvCYP707A1, FvCYP707A2 and FvCYP707A4) had chloroplast-targeting signal peptides. There were no P450 with mitochondrial-targeting signal peptides.

**Table 1:** List of full-length *Fontainea picrosperma P450s* identified in this study. Cellular location of the protein predicted using the TargetP program. ‘C’: chloroplast; ‘S’: secreted; ‘*’: unknown and ‘-’ not secreted

Gene name	Length	Type	Family	Subfamily	Instability index (ii)	Clan	PI	Mol. Wt (kDa)	Loc
**FpCYP727B1**	360	Non-A	CYP727	CYP727B	33.54	CYP727	8.26	40.4	–
**FpCYP97C1**	552	Non-A	CYP97	CYP97C	33.6	CYP97	7.57	61.92	*
**FpCYP94A1**	499	Non-A	CYP94	CYP94A	42.39	CYP86	6.79	56.97	S
**FpCYP86A1**	545	Non-A	CYP86	CYP86A	45.8		8.4	62.07	S
FpCYP86A2*	528	Non-A	CYP86	CYP86A	46.42		7.55	60.38	S
**FpCYP96A1**	334	Non-A	CYP96	CYP96A	48.86		8.75	39.16	S
FpCYP711A1*	393	Non-A	CYP711	CYP711A	32.09	CYP711	6.1	44.82	S
FpCYP711A2*	529	Non-A	CYP711	CYP711A	35.1		8.72	60.64	S
**FpCYP714A1**	535	Non-A	CYP714	CYP714A	41.82	CYP72	9.14	61.25	S
**FpCYP714A2**	426	Non-A	CYP714	CYP714A	37.63		9.12	48.55	*
**FpCYP714A3**	353	Non-A	CYP714	CYP714A	40.39		8.68	40.66	–
FpCYP714A4*	529	Non-A	CYP714	CYP714A	39.97		9.08	60.3	S
FpCYP709F*	519	Non-A	CYP709	CYP709F	32.92		7.93	59.96	S
FpCYP709F1*	423	Non-A	CYP709	CYP709F	41.98		8.93	48.35	–
FpCYP709F2*	523	Non-A	CYP709	CYP709F	41.16		9.44	59.31	S
**FpCYP734A1**	520	Non-A	CYP734	CYP734A	44.48		8.81	59.34	S
FpCYP734A3*	510	Non-A	CYP734	CYP734A	35.42		8.29	58.52	S
**FpCYP721A1**	368	Non-A	CYP721	CYP721A	44.48		9.28	42.38	S
**FpCYP721A2**	499	Non-A	CYP721	CYP721A	39.12		9.24	57.06	S
**FpCYP721A3**	429	Non-A	CYP721	CYP721A	45.73		8.45	49.11	S
**FpCYP721A5**	334	Non-A	CYP721	CYP721A	40.28		8.68	38.73	S
FpCYP721A6*	512	Non-A	CYP721	CYP721A	40.94		9.2	58.25	S
FpCYP721A7*	332	Non-A	CYP721	CYP721A	43.55		7.07	38.84	–
**FpCYP72A1**	440	Non-A	CYP72	CYP72A	42.58		9.2	50.01	S
FpCYP72A2*	405	Non-A	CYP72	CYP72A	39.66		9.58	46.29	S
**FpCYP51G**	485	Non-A	CYP51	CYP51G	36.21	CYP51	6.96	55.62	*
**FpCYP710A1**	504	Non-A	CYP710	CYP710A	41.32	CYP710	9.13	57.69	S
**FpCYP74A1**	521	Non-A	CYP74	CYP74B	51.19	CYP74	8.74	58.38	C
**FpCYP74B1**	496	Non-A	CYP74	CYP74B	51.66		8.55	55.67	C
**FpCYP74B2**	492	Non-A	CYP74	CYP74B	51.86		8.13	55.25	C
**FpCYP707A2**	440	Non-A	CYP707	CYP707A	40.71	CYP85	9.12	50.5	S
**FpCYP707A3**	470	Non-A	CYP707	CYP707A	43.31		9.23	53.69	S
**FpCYP707A4**	489	Non-A	CYP707	CYP707A	41.31		9.04	56.05	*
**FpCYP88A1**	479	Non-A	CYP88	CYP88A	40.55		9.06	54.7	S
**FpCYP88A2**	482	Non-A	CYP88	CYP88A	40.16		8.65	55.99	S
**FpCYP88A3**	489	Non-A	CYP88	CYP88A	44.99		9.15	56.9	S
FpCYP88A4*	446	Non-A	CYP88	CYP88A	44.33		8.92	51.07	S
FpCYP733A1*	324	Non-A	CYP733	CYP733A	34.08		8.52	36.53	S
FpCYP733A2*	476	Non-A	CYP733	CYP733A	39.44		9.11	54.74	S
FpCYP733A3*	414	Non-A	CYP733	CYP733A	40.75		9.28	47.7	*
FpCYP728D1*	326	Non-A	CYP728	CYP728D	40.59		9.75	37.57	*
**FpCYP728D2**	476	Non-A	CYP728	CYP728D	42.7		9.43	54.78	*
**FpCYP716E1**	410	Non-A	CYP716	CYP716E	35.24		9.07	46.71	*
**FpCYP90D1**	514	Non-A	CYP90	CYP90D	50.04		9.57	58.06	*
**FpCYP90B1**	494	Non-A	CYP90	CYP90B	46.45		8.87	56.62	S
**FpCYP85A1**	464	Non-A	CYP85	CYP85A	40.53		9.25	53.25	S
**FpCYP93B1**	512	A	CYP93	CYP93B	30.25	CYP71	8.72	59.62	S
**FpCYP93D1**	514	A	CYP93	CYP93D	35.84		6.52	57.99	S
**FpCYP712C1**	534	A	CYP712	CYP712C	36.06		8.21	60.27	*
FpCYP82C2*	362	A	CYP82	CYP82C	38.11		9.02	41.89	*
FpCYP82C3*	528	A	CYP82	CYP82C	36.1		8.66	60.29	*
FpCYP82J1*	520	A	CYP82	CYP82J	35.58		6.97	58.78	S
**FpCYP82C5**	320	A	CYP82	CYP82C	23.68		5.64	35.19	*
FpCYP82D1*	306	A	CYP82	CYP82D	34.39		9.8	27.7	S
FpCYP82D3*	407	A	CYP82	CYP82D	41.37		6.24	46.64	*
FpCYP82C6*	519	A	CYP82	CYP82C	38.41		8.1	59.04	S
**FpCYP82C7**	310	A	CYP82	CYP82C	47.75		9.33	35	*
**FpCYP82C8**	510	A	CYP82	CYP82C	43.49		8.51	57.69	*
**FpCYP76D1**	517	A	CYP76	CYP76D	50.71		8.75	58.09	S
**FpCYP81T1**	308	A	CYP81	CYP81T	42.27		9.52	35.93	S
**FpCYP81T2**	496	A	CYP81	CYP81K	43.06		8.73	56.37	S
**FpCYP81K1**	505	A	CYP81	CYP81K	46.38		7.15	57.23	S
**FpCYP81K2**	336	A	CYP81	CYP81K	52.28		9.17	38.76	S
**FpCYP76D2**	498	A	CYP76	CYP76D	49.8		8.94	57.4	S
FpCYP76D3*	316	A	CYP76	CYP76D	50.45		7.63	36.54	S
FpCYP76D4*	499	A	CYP76	CYP76D	52.65		8.26	57.6	S
**FpCYP81S1**	507	A	CYP81	CYP81S	53.39		8.17	58.4	S
**FpCYP706C1**	517	A	CYP706	CYP706C	37.2		7.21	57.32	S
FpCYP76C1*	310	A	CYP76	CYP76C	39.16		5.22	35.76	*
**FpCYP76C2**	495	A	CYP76	CYP76C	37.54		6.62	56.57	S
**FpCYP76C3**	304	A	CYP76	CYP76C	45.99		8.81	34.15	S
**FpCYP76C4**	309	A	CYP76	CYP76C	42.45		7.58	34.74	S
FpCYP76C5*	373	A	CYP76	CYP76C	42.04		9.27	42.75	S
FpCYP76D5*	300	A	CYP76	CYP76D	40.34		9.02	27.22	S
FpCYP76D6*	504	A	CYP76	CYP76D	42.06		7.92	57.97	S
**FpCYP92A1**	444	A	CYP92	CYP92D	28.16		5.84	50.71	*
FpCYP84A1*	405	A	CYP84	CYP84A	43.34		7.06	46.46	S
**FpCYP84A2**	513	A	CYP84	CYP84A	45.24		6.36	58.83	S
**FpCYP71A1**	498	A	CYP71	CYP71A	37.17		8.52	56.03	*
**FpCYP71A2**	431	A	CYP71	CYP71A	35.72		6.37	49.77	S
**FpCYP71A3**	498	A	CYP71	CYP71A	37.17		8.52	56.03	*
**FpCYP83**	496	A	CYP83		31.08		8.92	56.1	S
FpCYP71B3*	464	A	CYP71	CYP71B	46.97		9	53.29	*
FpCYP71B4*	513	A	CYP71	CYP71B	45.36		8.59	58.78	S
FpCYP71B5*	311	A	CYP71	CYP71B	40.63		9.14	35.62	S
**FpCYP71B6**	329	A	CYP71	CYP71B	46.63		9.45	37.42	S
**FpCYP71D1**	497	A	CYP71	CYP71D	40.04		8.51	56.89	S
FpCYP71B10*	519	A	CYP71	CYP71B	40.55		8.57	59.1	S
**FpCYP726A1**	501	A	CYP726	CYP726A	38.85		8.66	56.38	S
**FpCYP726A2**	498	A	CYP726	CYP726A	38.84		8.72	56.11	S
FpCYP726A3*	512	A	CYP726	CYP726A	39.99		8.58	58.16	S
FpCYP726A4*	614	A	CYP726	CYP726A	41.58		6.56	68.87	C
FpCYP71D3*	395	A	CYP71	CYP71D	46.46		9.26	44.82	S
FpCYP71B12*	342	A	CYP71	CYP71B	36.6		9.12	37.94	C
**FpCYP78A3**	528	A	CYP78	CYP78A	28.71		9.11	59.95	*
**FpCYP78A4**	509	A	CYP78	CYP78A	39.21		8.89	58.15	S
**FpCYP78A5**	535	A	CYP78	CYP78A	39.98		6.3	60.07	*
**FpCYP73A1**	505	A	CYP73	CYP73A	44.78		9.1	58.36	S
**FpCYP701A1**	408	A	CYP701	CYP701A	42.79		6.71	46.77	*
**FpCYP77A1**	502	A	CYP77	CYP77A	39.35		8.96	57.61	S
**FpCYP89A1**	508	A	CYP89	CYP89A	52.37		8.51	58.45	S
**FpCYP89A2**	517	A	CYP89	CYP89A	52.46		7.69	59.06	S
**FpCYP89A3**	534	A	CYP89	CYP89A	60.59		8.91	61.38	*

**Table 2:** List of full-length *Fontainea venosa P450s* identified in this study. Cellular location of the protein predicted using the TargetP program. ‘C’: chloroplast; ‘S’: secreted; ‘*’: unknown and ‘-’ not secreted

Gene name	Length	Type	Family	Subfamily	Instability index (ii)	Clan	PI	Mol. Wt (kDa)	Loc
FvCYP97B1*	391	Non-A	CYP97	CYP97B	46.12	CYP97	6.63	44.46	–
FvCYP97B2*	587	Non-A	CYP97	CYP97B	43.71		8.04	66.16	*
FvCYP97A*	632	Non-A	CYP97	CYP97A	43.34		6.1	70.76	*
FvCYP97A1*	419	Non-A	CYP97	CYP97A	46.66		5.29	47.55	*
FvCYP97A2*	441	Non-A	CYP97	CYP97A	41.18		6.2	50.47	*
**FvCYP97C1**	566	Non-A	CYP97	CYP97C	32.16		8.24	63.23	*
FvCYP704A1*	520	Non-A	CYP704	CYP704A	41.27	CYP86	6.32	60.1	S
FvCYP704A2*	457	Non-A	CYP704	CYP704A	41.94		5.43	53.17	S
FvCYP704A3*	390	Non-A	CYP704	CYP704A	41.62		8.48	44.12	*
FvCYP704A4*	516	Non-A	CYP704	CYP704A	39.07		8.18	59.67	S
FvCYP94D1*	503	Non-A	CYP94	CYP94D	38		7	58.19	S
**FvCYP94A1**	499	Non-A	CYP94	CYP94A	40.16		8.18	56.37	S
FvCYP94C1*	452	Non-A	CYP94	CYP94C	47.09		9.42	51.82	S
FvCYP94B1*	514	Non-A	CYP94	CYP94B	42.88		8.95	59.52	S
**FvCYP86A1**	545	Non-A	CYP86	CYP86A	45.31		8.65	62.02	S
FvCYP86B1*	550	Non-A	CYP86	CYP86B	41.66		8.74	63.41	–
**FvCYP96A1**	333	Non-A	CYP96	CYP96A	49.51		8.26	39.24	S
**FvCYP727B1**	550	Non-A	CYP72	CYP727B	33.13	CYP727	8.7	62.33	–
**FvCYP727B2**	548	Non-A	CYP72	CYP727B	32.14		8.24	62.36	–
FvCYP714E1*	382	Non-A	CYP714	CYP714E	38.03	CYP72	9.32	58.57	S
FvCYP714E2*	515	Non-A	CYP714	CYP714E	35.26		9.2	43.72	S
**FvCYP714A1**	549	Non-A	CYP714	CYP714A	39.3		9.28	62.67	S
FvCYP714A5*	512	Non-A	CYP714	CYP714A	36.34		9.36	58.8	S
**FvCYP734A1**	520	Non-A	CYP734	CYP734A	45.3		8.49	59.34	S
**FvCYP734A2**	349	Non-A	CYP734	CYP734A	46.25		6.02	39.75	*
FvCYP734A4*	360	Non-A	CYP734	CYP734A	38.02		8.74	40.24	*
FvCYP734A5*	526	Non-A	CYP734	CYP734A	41.86		9.59	60.09	S
**FvCYP721A1**	499	Non-A	CYP721	CYP721A	43.06		9.14	57.1	S
**FvCYP721A3**	512	Non-A	CYP721	CYP721A	42.85		9.14	58.1	S
**FvCYP721A4**	403	Non-A	CYP721	CYP721A	45.12		9.17	46.32	S
**FvCYP721A5**	335	Non-A	CYP721	CYP721A	42.39		8.48	38.76	*
FvCYP721A8*	369	Non-A	CYP72	CYP721A	48.81		9.61	42.9	S
FvCYP721A9*	311	Non-A	CYP72	CYP721A	51.09		7.7	36.23	S
FvCYP72D1*	519	Non-A	CYP72	CYP72D	32.21		8.02	59.24	S
FvCYP72D2*	355	Non-A	CYP72	CYP72D	38.3		5.53	40.77	–
**FvCYP72A1**	505	Non-A	CYP72	CYP72A	45.33		9.21	58.87	S
**FvCYP51G**	485	Non-A	CYP51	CYP51G	36.63	CYP51	7.72	55.74	S
**FvCYP710A1**	411	Non-A	CYP710	CYP710A	41.13	CYP710	8.68	46.98	*
**FvCYP74A1**	517	Non-A	CYP74	CYP74A	48.45	CYP74	8.07	57.45	C
**FvCYP74B1**	496	Non-A	CYP74	CYP74B	51.46		8.91	55.81	C
**FvCYP74B2**	492	Non-A	CYP74	CYP74B	50.35		7.63	55.2	C
FvCYP707A1*	347	Non-A	CYP707	CYP707A	30.45	CYP85	9.27	39.89	S
**FvCYP707A2**	553	Non-A	CYP707	CYP707A	43.04		9.23	63.52	*
**FvCYP707A4**	463	Non-A	CYP707	CYP707A	40.45		9.05	52.43	S
**FvCYP88A1**	479	Non-A	CYP88	CYP88A	40.52		9.09	54.67	S
**FvCYP88A3**	490	Non-A	CYP88	CYP88A	41.57		9.05	56.76	S
**FvCYP85A1**	464	Non-A	CYP85	CYP85A	41.83		9.23	53.3	S
FvCYP722A1*	476	Non-A	CYP722	CYP722A	41.48		9.13	54.67	S
**FvCYP728D2**	478	Non-A	CYP728	CYP728D	41.32		9.46	54.05	*
**FvCYP716E1**	410	Non-A	CYP716	CYP716E	38.77		8.92	47.87	*
FvCYP716C1*	403	Non-A	CYP716	CYP716C	44.65		8.92	46.21	*
FvCYP90A1*	473	Non-A	CYP90	CYP90A	41.04		9.18	54.75	*
**FvCYP90D1**	547	Non-A	CYP90	CYP90D	47.54		9.55	63.9	*
**FvCYP90D2**	360	Non-A	CYP90	CYP90D	40.74		9.09	41.2	*
**FvCYP90B1**	494	Non-A	CYP90	CYP90B	47.54		8.86	56.73	S
FvCYP79D1*	524	A	CYP79	CYP79D	37.27	CYP71	9.22	59.35	S
FvCYP79D2*	526	A	CYP79	CYP79D	37.18		5.49	35.34	S
**FvCYP93B1**	512	A	CYP93	CYP93B	30.31		8.61	58.18	S
**FvCYP93D1**	514	A	CYP93	CYP93D	36.8		6.12	57.95	S
FvCYP93A1*	515	A	CYP93	CYP93A	35.37		6.39	58.08	S
**FvCYP712C1**	503	A	CYP712	CYP712C	30.73		7.98	56.85	S
FvCYP712E1*	349	A	CYP712	CYP712E	42.4		5.58	40.66	*
FvCYP82C4*	520	A	CYP82	CYP82C	38.44		8.83	59.86	S
FvCYP82J2*	360	A	CYP82	CYP82J	34.04		9.29	40.5	S
FvCYP82J3*	455	A	CYP82	CYP82J	38.56		6.74	51.89	–
**FvCYP82C5**	319	A	CYP82	CYP82C	21.55		5.95	35.37	*
FvCYP82D2*	320	A	CYP82	CYP82D	43.94		6.61	36.06	*
**FvCYP82C7**	514	A	CYP82	CYP82C	49.83		7.61	58.84	S
**FvCYP76D1**	517	A	CYP76	CYP76D	52.75		8.62	58.98	S
**FvCYP81T1**	307	A	CYP81	CYP81T	45.32		9.52	35.76	S
**FvCYP81T2**	496	A	CYP81	CYP81T	47.83		8.85	56.28	S
FvCYP81D1*	501	A	CYP81	CYP81D	47.78		8.9	57.91	*
FvCYP81D2*	514	A	CYP81	CYP81D	45.65		8.6	59.53	S
**FvCYP81K1**	505	A	CYP81	CYP81K	44.71		8.22	57.46	S
**FvCYP81K2**	322	A	CYP81	CYP81K	51.8		9.13	37.8	S
**FvCYP76D2**	498	A	CYP76	CYP76D	48.92		8.39	56.22	S
**FvCYP81S1**	504	A	CYP81	CYP81S	54.93		8.34	57.07	S
FvCYP81S2*	306	A	CYP81	CYP81S	52.02		9.05	35.19	S
**FvCYP706C1**	517	A	CYP706	CYP706C	37.72		8.37	57.22	S
FvCYP76H1*	503	A	CYP76	CYP76H	38.56		8.69	57.06	S
FvCYP76I1*	509	A	CYP76	CYP76I	30.36		9.11	58.12	S
**FvCYP76C2**	495	A	CYP76	CYP76C	41.51		6.88	56.79	S
FvCYP76D8*	493	A	CYP76	CYP76D	41.85		6.16	55	S
FvCYP76D7*	494	A	CYP76	CYP76D	37.17		6.19	55.01	S
**FvCYP92A1**	330	A	CYP92	CYP92A	26.99		9.22	37.7	*
**FvCYP84A2**	499	A	CYP84	CYP84A	45.27		6.07	56	*
FvCYP71B11*	526	A	CYP71	CYP71B	38.06		8.53	60.37	S
FvCYP71B2*	342	A	CYP71	CYP71B	45.86		8.64	39.28	S
FvCYP71*	516	A	CYP71		36.06		6.83	59.34	*
FvCYP71AU1*	311	A	CYP71	CYP71AU	29.54		6.97	35.94	S
FvCYP71AU2*	500	A	CYP71	CYP71AU	32.65		6.15	56.48	S
**FvCYP71A1**	499	A	CYP71	CYP71A	35.27		8.03	57.42	*
FvCYP83B1*	498	A	CYP83	CYP83B	42.49		9.07	57.05	S
FvCYP83B2*	497	A	CYP83	CYP83B	39.52		8.65	57.59	S
**FvCYP83**	339	A	CYP83		34.22		9.91	38.67	S
FvCYP71C1*	506	A	CYP71	CYP71C	36.92		7.65	57.95	S
FvCYP71C2*	305	A	CYP71	CYP71C	31.29		8.26	34.31	S
FvCYP71AP*	511	A	CYP71	CYP71AP	47.75		5.92	58.97	S
FvCYP71B12*	301	A	CYP71	CYP71B	43.28		9.69	27.62	S
FvCYP71B13*	506	A	CYP71	CYP71B	39.73		9.04	58.87	S
**FvCYP71B6**	349	A	CYP71	CYP71B	47.68		9.56	40.18	S
**FvCYP71D1**	498	A	CYP71	CYP71D	40		8.32	56.18	S
FvCYP71B7*	514	A	CYP71	CYP71B	43.43		8.73	58.25	S
FvCYP71B8*	320	A	CYP71	CYP71B	49.73		9.22	36.59	S
FvCYP71B9*	343	A	CYP71	CYP71B	37.29		6.28	38.62	S
**FvCYP726A1**	501	A	CYP726	CYP726A	37.98		8.8	56.33	*
FvCYP71D2*	503	A	CYP71	CYP71D	43.37		7.71	56.75	S
FvCYP71B11*	548	A	CYP71	CYP71B	38.06		8.53	60.37	*
FvCYP98A1*	508	A	CYP98	CYP98A	39.14		7.77	57.96	S
FvCYP78A1*	538	A	CYP78	CYP78A	29.08		8.38	60.6	S
FvCYP78A2*	468	A	CYP78	CYP78A	39.04		8.93	52.54	–
**FvCYP78A3**	528	A	CYP78	CYP78A	29.5		8.93	59.96	*
**FvCYP78A4**	514	A	CYP78	CYP78A	37.91		9.16	58.96	*
**FvCYP78A5**	534	A	CYP78	CYP78A	38.41		6.26	60.03	S
**FvCYP73A1**	505	A	CYP73	CYP73A	45.67		9.11	58.38	*
**FvCYP701A1**	514	A	CYP701	CYP701A	43.82		7.62	58.51	S
**FvCYP701A2**	408	A	CYP701	CYP701A	45.2		6.15	46.67	*
FvCYP77B1*	505	A	CYP77	CYP77B	40		7.53	57.06	*
**FvCYP77A1**	502	A	CYP77	CYP77A	37.73		9.29	57.89	S
**FvCYP89A1**	514	A	CYP89	CYP89A	51.56		8.51	59.23	S
**FvCYP89A2**	338	A	CYP89	CYP89A	49.41		5.64	39.59	S
**FvCYP89A3**	520	A	CYP89	CYP89A	54.72		8.78	59.54	*
**FvCYP89A4**	517	A	CYP89	CYP89A	49.02		8.77	59.14	*

### Phylogenetic and putative functional analysis of P450

**Fig. 2:**
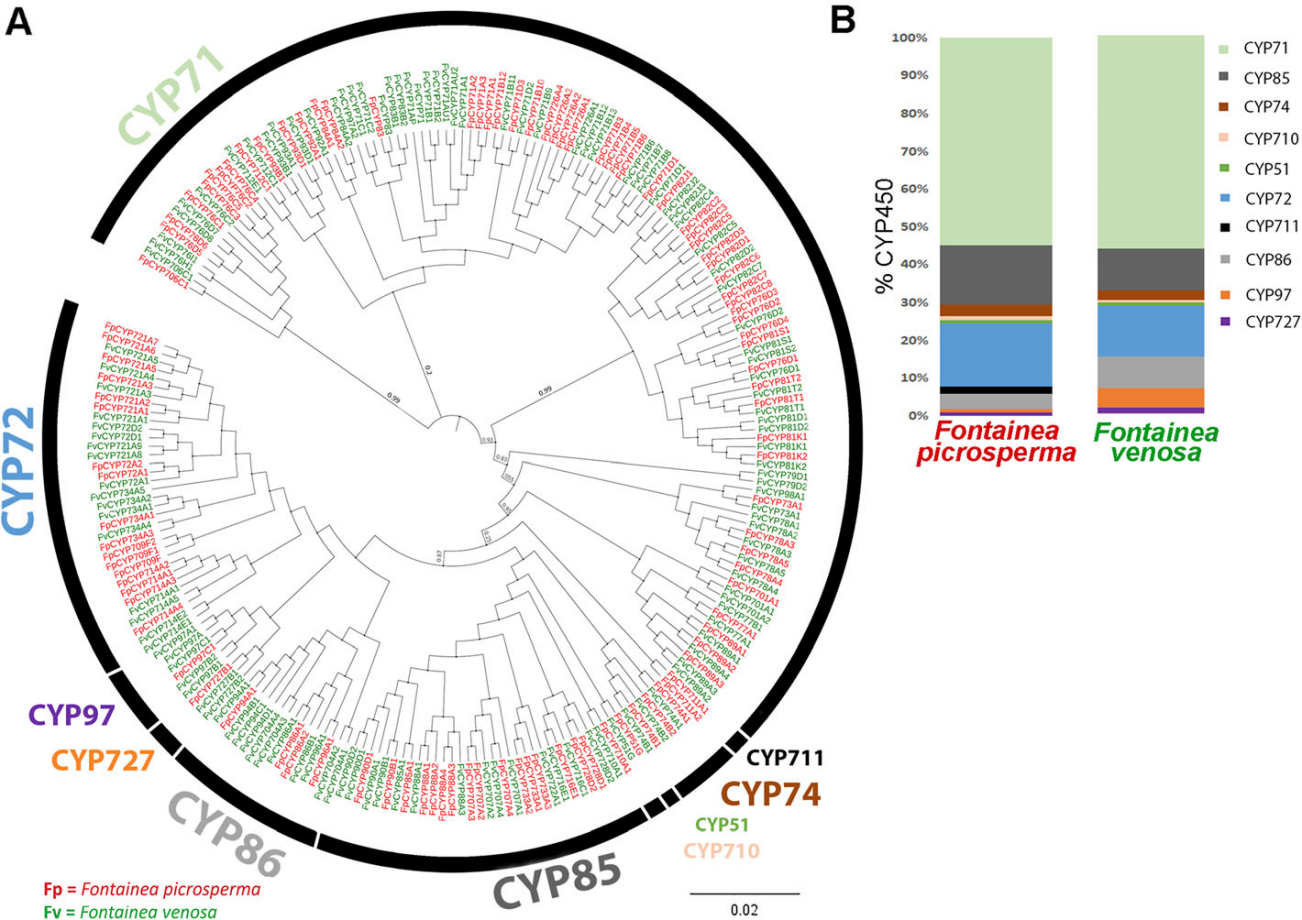
Phylogenetic and putative functional analysis of P450 in *Fontainea picrosperma* and *Fontainea venosa*, including their clans*.* **a** Maximum likelihood tree constructed using FastTree in Geneious. Asterisks represent P450 unique to each *Fontainea* species (percentage of identity 92%). The branch length represents genetic distance and the value on the branch is the support rate. **b** Distribution of P450 in *F. picrosperma* and *F. venosa* based on clans. CYP71, alkaloid, sesquiterpenoids, cyclic terpenoid and flavonoid synthesis; CYP74, synthesis of oxylipin derivatives and allene oxide in the octadecanoid and jasmonate pathways; CYP51, regulates the synthesis of sterols and triterpenes; CYP85, modification of cyclic terpenes and sterols in the brassinosteroid (BR), abscisic acid (ABA) and gibberellin (GA) pathways; CYP97, hydroxylation of carotenoids; CYP72, catabolism of isoprenoid hormones CYP711 are identified as a strigolactones biosynthetic enzyme. The functions of CYP727 are currently unknown

A phylogenetic analysis containing 1042 P450s from 6 species (*F. picrosperma, F. venosa, R. communis, M. esculenta, A. thaliana* and *S. lycopersicum*) confirmed that the majority of *F. picrosperma* and *F. venosa* P450 do not show substantial relatedness to P450 from species outside of the Euphorbiaceae family (Additional Fig. 2). There were 72 *P450* genes exclusive to *Fontainea* (identity > 92%). A *Fontainea*-specific P450 phylogeny showed that in *F. picrosperma,* 16 CYP85 were assigned into 7 families that form a single clade, while the CYP72 clan contained 17 genes assigned to 3 families **(**Fig. 2a**)**. In *F. venosa,* 14 genes were assigned into 7 families that formed a single clade for CYP85. In the CYP72 clade, 17 P450 clustered into 4 families. A single P450 was represented in clan CYP710 (FpCYP710A1, FvCYP710A1) and CYP51 (FpCYP51G, FvCYP51G), which are phylogenetically most related to CYP85.

The majority of *F. picrosperma* P450 belong to the CYP71 clan (57 genes; 57.68%), followed by the CYP72 and CYP85 clans, which is also known as A-type **(**Fig. 2b**).** The CYP71 clan is responsible for alkaloid, sesquiterpenoid, cyclic terpenoid and flavonoid biosynthesis. The majority of *F. venosa* P450 also belong to the CYP71 clan (68 genes; 55.28%), followed by the CYP72 and CYP85 clans **(**Fig. 2b**)**. The non-A types encompass the remaining 46 P450, which belong to 9 P450 clans and 21 families in *F. picrosperma*. In *F. venosa,* there were 55 non-A type P450, which belong to 8 clans and 19 families. Of note, representative P450 from CYP711 were absent from *F. venosa*, while CYP97 were more well represented in *F. venosa* compared to *F. picrosperma*.

### Comparative expression of *P450* genes between *Fontainea* species

**Fig. 3:**
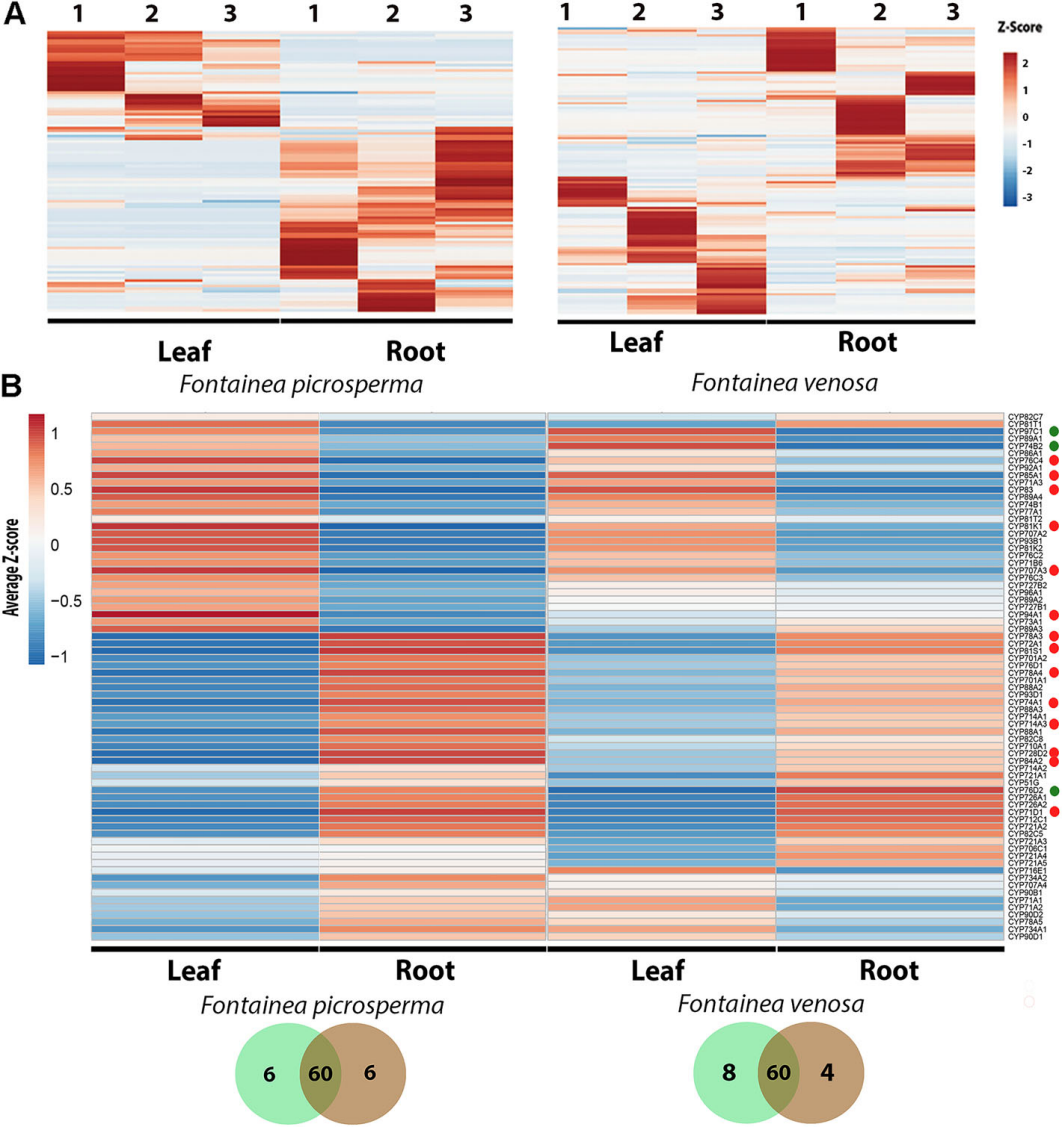
*Fontainea P450* expression analysis. **a** Expression profiles of 103 and 123 full-length P450 genes in *Fontainea picrosperma* and *Fontainea venosa*, respectively, based on RNA-seq experiment in 3 different leaf and root samples. See Additional File for list of genes. **b** Homolog P450 average z-score value expression profile in *Fontainea* species. Red circle, represents significantly different (*p* < 0.05) between root and leaf tissues in *F. picrosperma*. Green circle represents significantly different (*p* < 0.05) between root and leaf tissues in *F. venosa.* See Additional Fig. 4 for significance values. Venn diagrams shown below indicates those P450 exclusive and shared between leaf and root tissues

Overall *P450* expression patterns demonstrated that, in both species, root tissue had higher expression (66.2% *F. picrosperma,* 65.0% *F. venosa*), compared to leaf tissue **(**Fig. 3a). An expression profile heatmap of the 72 homologous *P450* genes, common to both species of *Fontainea,* showed that the majority of genes were more active in root tissue (44.4%) compared to leaf tissue (25.0%) **(**Fig. 3b**)**. In *F. picrosperma*, there were 6 *P450* genes exclusively expressed in leaf tissue (CYP82C7, CYP81T1, CYP89A3, CYP73A1, CYP94A1 and CYP727B1) and 6 exclusive to the root tissue (CYP90D1-D2, CYP78A5, CYP71A1-A2, CYP707A4). Also in *F. picrosperma*, 8 *P450* were significantly more highly expressed in root tissue, 6 genes were more highly expressed in leaf issue. In *F. venosa*, 8 exclusive to leaf tissue (CYP90D1-D2, CYP734A1, CYP78A5, CYP71A1-A2, CYP707A4 and CYP716E1) and 4 *P450* genes were exclusively expressed in root tissue (CYP82C7, CYP89A3, CYP73A1 and CYP81T1) **(**Fig. 3b**)**. Only 2 *P450* genes were significantly more highly expressed in leaf tissue and one gene significantly more highly expressed in root tissue.

### *P450* gene candidates involved in diterpenoid metabolism

**Fig. 4:**
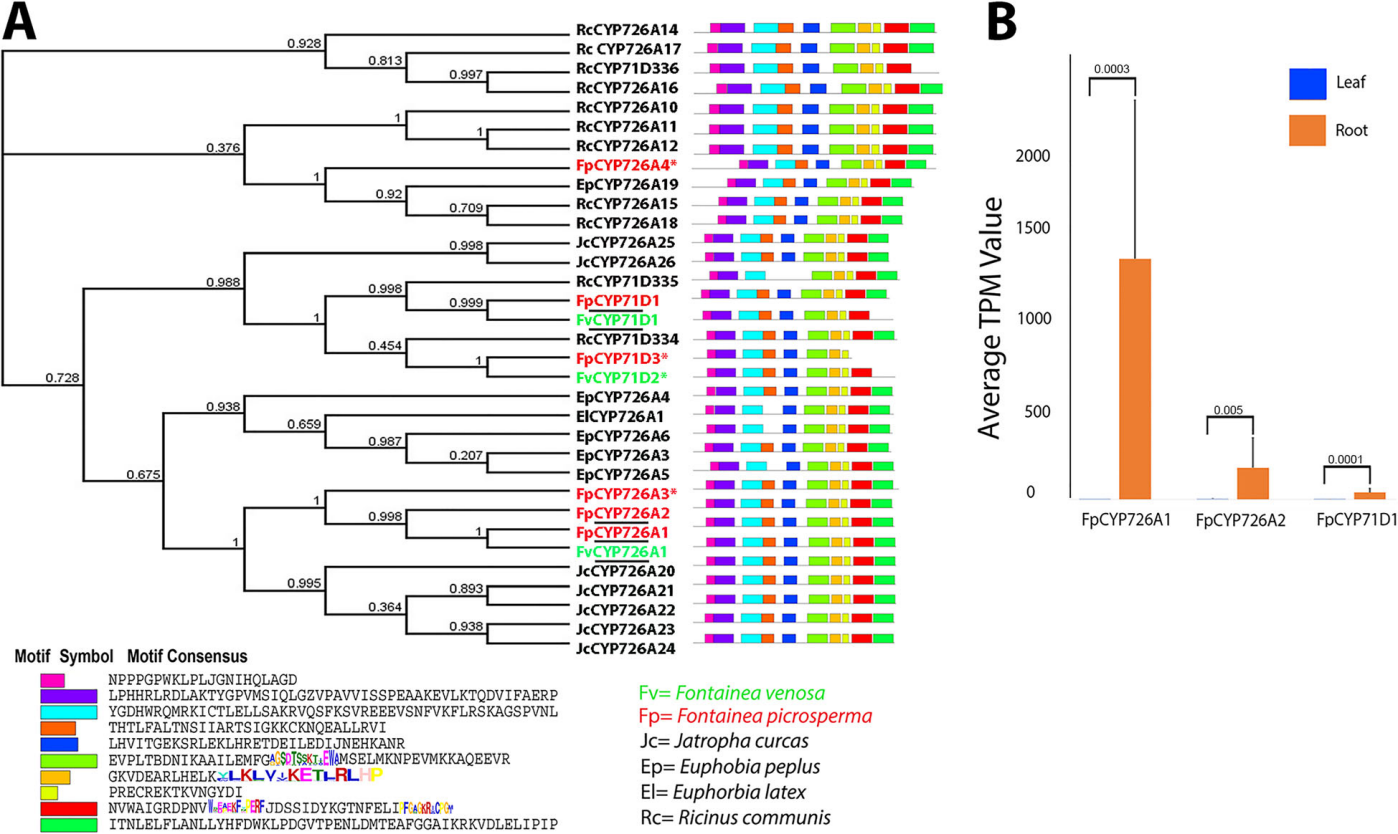
Phylogenetic analysis of diterpenoid CYP genes in *Fontainea* species. **a** Phylogenetic presentation of *Fontainea* species-specific CYP genes with other Euphorbiaceae and their motif analysis and the four conserved motif region and one unique conserved motif in diterpenoids represented by weblogo. Underlined genes are present in both *Fontainea* species and asterisk genes are species-specific (percentage of identity 92%). **b** Graph showing gene expression of diterpenoid genes across 12 biological replicates of *Fontainea picrosperma* in leaf and root tissue. Significant differences: *p* < 0.01. TPM, transcripts per million

Phylogenetic analysis of the *Fontainea* P450 classified within clans CYP71D and CYP726A were found to be closely related to those found in other Euphorbiaceae species (*Jatropha curcus*, *Euphorbia peplus*, *Euphorbia latex* and *R. communis*), confirming their position within these putative diterpenoid P450 subfamilies **(**Fig. 4a**)**. This was additionally supported by an expanded conserved motif analysis (in addition to I-helix, K-helix, PERF and heme-binding motifs). Of the 4 *F. picrosperma* CYP726A, FpCYP726A4 was most divergent. A single CYP726A was identified in *F. venosa* (FvCYP726A1), which formed a clade with FpCYP726A1-A2. Three of the *Fontainea* diterpenoid P450 were present in both *F. picrosperma* and *F. venosa* but not in other Euphorbiaceae.

To investigate tissue expression of the common *Fontainea* diterpenoid *P450* genes CYP726A1, CYP726A2 and CYP71D1, an additional 9 biological samples of *F. picrosperma* were quantitatively analysed (12 in total) by mapping RNA-seq to the reference transcriptome. These data showed statistically significantly higher levels of gene expression for all 3 genes in the root tissue compared to leaf tissue **(**Fig. 4b**,** Additional File 3). The housekeeping genes, glyceraldehyde-3-P dehydrogenase (GAPC) and elongation factor 1-alpha (EF1α) showed consistency in both leaf and root tissues and higher expression in root tissues compared to leaf tissue [12].

## Discussion

Cytochrome P450s are evolutionarily conserved enzymes that are involved in the catalysis of numerous reactions, required for growth, development, defence [34] and secondary metabolism [14]. Prior to this study, no *P450* genes had been identified, let alone characterized, in any species of the genus *Fontainea.* This is significant as *P450* genes are likely to be important for future understanding of the biosynthetic pathways that produce medicinally significant diterpene esters, such as TT, which are unique to *Fontainea*. Towards that aim, we have identified and classified putative full-length *P450* encoding genes in two species of *Fontainea*, *F. picrosperma* and *F. venosa*. Phylogenetic analysis allowed us to identify groups of genes for further evaluation. Moreover, their expression profiles in leaf and root tissues were investigated, with a particular focus on the *P450* genes linked with diterpenoid biosynthesis, potentially involved in the production of TT.

We report 103 and 123 full-length *P450* genes from *F. picrosperma* and *F. venosa,* respectively, that were classified into clans, which cumulatively consisted of 37 families and 67 subfamilies that fit into conformed plant-derived functions, most prominently with diterpenoid, flavonoid and other functions. An ortholog comparison showed that P450 genes of *Fontainea* species are largely unique when compared to other plant species of both Euphorbiaceae and non-Euphorbiaceae. In support of our metabolomics analysis, the *S. lycopersicum* and *A. thaliana* P450 showed low overall similarity to *Fontainea* species. This may be attributed to the unique biosynthesis of diterpenoid derivatives that are phorbol ester-specific, found in *Fontainea* and other members of the Euphorbiaceae family.

The total number of *Fontainea P450* identified in this study was consistent with that found in other plant transcriptomics *P450* research, including the total number of full-length sequences, clans, families and subfamilies [30, 35, 36]. For example, transcriptomic studies allowed the elucidation of 151 full-length *P450* genes in *Lonicera japonica* [35], 118 full-length in *Taxus chinensis* [36] and 116 full-length in *Salvia miltiorrhiza* [30]. However, in *S. miltorrhiza*, the tissues used for transcriptomics included leaves, roots and flowers, while in *L. japonica,* flower and buds were used. Our study identified 127 and 125 partial-length *P450* gene from *F. picrosperma* and *F. venosa*, respectively. To obtain the full-length sequence, additional RNA-seq from the stem, flower and fruit would be helpful, as well as from different stages of development. In addition, this could be complemented by genome sequencing.

If a genome is available for a species, it does provide an alternate mechanism for *P450* gene identification, through genome-wide interrogation. In *A. thaliana*, this approach identified 246 genes that clustered into 9 clans and 47 families [20, 37], while a much larger number were identified from the soybean (*G. max*) and rice (*Japonica)* genomes, containing 332 and 355 *P450* genes, respectively [38]. Far fewer were present in the legume (*Medicago truncatula*), where 151 putative *P450* genes were identified, including 135 novel *P450* [39]. We expect that once a genome is available for *Fontainea*, a more complete list of full-length *P450* genes will be established.

Our results using these predictive protein characterisation analyses (i.e. molecular weight, cell localization, function) were in line with prior studies of P450 proteins. P450s are typically anchored on the surface of the endoplasmic reticulum [40] and some may target to the plastids or mitochondria [41]. It is common that animal CYPs are anchored to mitochondria, but there is no report of any plant CYP with mitochondrial localization [39] except maize, where 3 CYPs have been reported (*Zea mays*) [42]. In our study of *F. picrosperma* or *F. venosa,* no deduced P450 proteins were predicted to have mitochondrial targeting peptides. CYP74A1, CYP74B1 and CYP74B2 in *F. picrosperma* and *F. venosa* and CYP726A4 and CYP71B12 in *F. picrosperma* were found in the chloroplast. In other plants, such as *Triticum araraticum, Z. mays* all members of CYP74 and CYP701 were targeted to chloroplast [42].

The diversity of different P450s between *F. picrosperma* and *F. venosa*, and other species, likely contributes to the observed differences in their chemical profiles, including diterpenes [25]. In all plant species that have been researched to date, the largest P450 clan is CYP71 [21]. The families and subfamilies within the clan have diverged remarkably during plant evolution, many of which are known to be involved in secondary metabolite biosynthesis of flavonoids and alkaloids [43]. Similarly, the CYP71 family is the largest P450 clan in *Fontainea* (see Fig. 2). On the contrary, two CYP711 representatives were identified from *F. picrosperma*, but were absent in *F. venosa,* although CYP711 have been described in other plant species [23]. In our phylogenetic tree, the CYP74 clan is adjacent to CYP711 family, suggesting that *Fontainea* CYP711 may also function within the metabolism of oxylipins and strigolactone signals [23], as strigolactones have been identified as branching inhibition hormones in plants, and several CYP711 have been experimentally confirmed as strigolactones biosynthetic enzymes [44, 45]. We additionally found that *F. venosa* had more *CYP97* genes compared to *F. picrosperma*; the CYP97 clan is involved in the hydroxylation of carotenoids [46]. Carotenoids are a group of widely distributed pigments derived from the ubiquitous isoprenoid biosynthetic pathway and play diverse roles in plant primary and secondary metabolism. Carotenoids contain two pigments, carotene and lutein, which absorb and transfer energy to protect chlorophyll [37]. We speculate that this may partially explain why *F. venosa* have darker leaves compared to *F. picrosperma*.

Based on other studies, P450 biosynthesis genes are relatively more highly expressed in root tissue compared to leaf tissue [25, 47–49]. In our study, we also found that *Fontainea* (*F. picrosperma* and *F. venosa*) *P450* genes were more highly expressed in root tissue compared to leaf tissue (see Fig. 3)*.* Among those significantly more highly expressed in the root (*n* = 3) have been associated with fertility reduction (CYP78A), UV stress tolerance (CYP84A), gibberellin metabolism (CYP714A) and jasmonic acid metabolism (CYP74A) [50–53]. Those significantly more highly expressed in the leaf include those previously associated with catalysing successive oxidation steps of the plant hormone jasmonoyl-isoleucine for catabolic turnover (CYP94), expression of ABA 8′-hydroxylase and affects ABA levels to control seed dormancy (CYP707A), hydroxylation of carotenoids (CYP97), biosynthesis of castasterone in the brassinosteroid biosynthetic pathway (CYP85A) and glucosinolate metabolism (CYP83) [40, 46, 54–56]. Some species variation existed in P450 homolog tissue expression (see Fig. 3b). This may be explained by the different growth and developmental stage of plants from which the tissue was sampled, as P450s are involved in the regulation of plant hormone metabolism, growth and development and hormones are involved in formation and development of flowers, leaves, stems and fruits [57].

Diterpenoids are one of the most widespread classes of secondary metabolites in higher plants, which are synthesized from basic isoprene units (C_5_H_8_) and further modified by various oxidoreductases, acyltransferases, dehydrogenases and glucosyltransferases [58]. P450-dependent oxidative modification is essential for the biosynthesis of diterpenes [58]. There are countless products formed in plants, among them diterpenoids are one of the most diverse groups, consisting of more than 12,000 metabolites [27] that have proven to be valuable as therapeutic drugs [59]. Our phylogenetic analysis of diterpenoid biosynthesis CYP71 clan members revealed that *Fontainea* have representatives within the CYP71D and CYP726A.

All *F*. *picrosperma* diterpenoid biosynthesis *P450* genes were significantly more highly expressed in root tissue compared to leaf tissue. The expression of genes can be affected by the developmental stage of plants, environmental conditions, seasonal and diurnal effects as well as biotic and abiotic stress [60]. Therefore, future research should explore the expression of the identified *P450* genes under these different scenarios and in additional tissues. In other plants, diterpenoid biosynthesis P450 genes were highly expressed in root tissue compared to leaf and flower [30]. Nonetheless, *Fontainea* CYP71D and CYP726A genes are excellent candidates for involvement in diterpenoid biosynthesis pathways, in particular, the biosynthesis of epoxy-tigliane diterpene esters, which are only found in species of *Fontainea,* although further experiments are required to confirm this hypothesis. Their identification allows for experimental analysis of their function, for example, in vitro expression of the proteins followed by gene expression detection, or by knock-in and knock-out can be completed. This may be followed by gene expression detection in vivo, depending on the availability of a robust experimental system. Also, the analysis of high TT producing *F. picroserma*, compared with low producers, will provide guidance about P450 (and other genes) that potentially regulate TT production.

## Conclusions

This research represents an important first step to understanding the role of *P450* genes associated with biosynthesis of diterpene esters in *Fontainea* species. A metabolome analysis showed that *Fontainea* species possess a chemical profile different from other plant species. This could at least partially be explained by the diversity of unique *P450* found in *Fontainea.* Further intra-genus chemical variation could be due to variation in *P450*, specifically those that are predicted to be involved in diterpenoid metabolism (CYP71D1, CYP726A1 and CYP726A2), which are significantly more highly expressed in the root tissue compare to leaf tissue. These P450 are strong candidates as key enzymes required for the biosynthesis of medicinally significant diterpene esters of the epoxy-tigliane class.

## Materials and methods

### Plant tissue collection for tigilanol tiglate analysis

Root, leaf, bark and fruit tissue samples were collected from *F. picrosperma* seedlings grown in the University of the Sunshine Coast (USC, Sippy Downs) greenhouse. Plants were grown in independent pots and kept in the greenhouse at ambient temperature and humidity according to Mitu et al. [12]. Dried (~ 150 mg) *F. picrosperma* root, bark, fruit and leaf tissues were extracted using methanol and the presence of TT confirmed using standard HPLC-UV techniques. Briefly, using an Agilent 1260 HPLC System, at 249 nm, with a Halo RP Amide 2.7 μm, 150 mm × 4.6 mm column and acetonitrile/water solvent scheme, UV profiles were compared to linear standard curves prepared using TT (supplied by EcoBiotics) between 0.0003 and 0.3 mg/mL.

### Metabolome analysis

Fresh mature leaves of *F. picrosperma, F. venosa, M. esculenta, R. communis, S. lycopersicum* and *A. thaliana* were collected from at least 2 individual plants. The leaves were cleaned and placed over silica-gel in zip-lock bags for 2 weeks in dark at room temperature. From 0.1 g of dried ground leaves, 3 mL of methanol was added. Ultrasound extraction was carried out for 1 min at room temperature prior to filtration onto a pre-weighed 20 mL borosilicate glass scintillation vial. The residue was extracted again with 3 mL of methanol in the same conditions. The methanol in the filtrate was evaporated using a Genevac centrifugal EZ-2 evaporator. The dried residue was then reconstituted in methanol to prepare a solution at 1 mg/mL and stored at − 20 °C in dark. Separation was performed on an ExionLC uHPLC system (Shimadzu) equipped with a Kinetex C_18_ column (2.1 × 150 mm, 100 Å). The column compartment was maintained at 40 °C and the auto-sampler was kept at 15 °C. The mobile phase was composed of water with 0.1% formic acid (v/v) (solvent A) and acetonitrile with 0.1% formic acid (v/v) (solvent B), and was run at a flow rate of 0.5 mL/min. The linear gradient started at 2% solvent B for 0.1 min, increased to 100% solvent B for 14.5 min and then was kept at this level for 1.4 min. Starting conditions were achieved in 0.5 min and re-equilibrated for 3.5 min, resulting in a total uHPLC run time of 20 min. The injection volume was 5 μL. The X500R QTOF mass spectrometer (Sciex) equipped with an ESI source in a positive mode was controlled by the Sciex OS software. The curtain gas, gas 1, gas 2 were set at 25, 40 and 50 psi, respectively. The spray voltage, declustering potential and collision energy were applied at 5500, 100 and 35 V, respectively. Spectral data was recorded in the mass range of *m/z* 100–1500 Da. The MS ions were extracted from LC-MS data using MZmine 2 (version 2.53) with setting parameters including retention time 0.6–17.5 min, retention time tolerance of 0.1 min, mass range *m/z* 100–1500 Da, mass tolerance of 0.02 Da, a noise threshold of 100. The pre-processed peak table data matrix was submitted onto a web-based service MetaboAnalyst (https://www.metaboanalyst.ca/), for metabolomic data analysis.

### Plant tissue collection for transcriptomics

*F. picrosperma* and *F. venosa* seedlings were provided by EcoBiotics Ltd. and plants were grown in the USC (Sippy Downs) greenhouse. Plants were grown in independent pots and kept in the greenhouse at ambient temperature and humidity according to Mitu et al. [12]. The plants were used from 2 to 4 years old and healthy, fully expanded leaves and actively growing root tips, including the apical meristem and root caps were dissected (single leaf from each plant) and (1 cm^2^ root) from plants of each species and preserved following the procedure described by Mitu et al. [12].

### RNA isolation

Total RNA was isolated from ~ 100 mg of leaf and root tissue of 12 individual *F. picrosperma* and 3 individual *F. venosa* using the Qiagen mini plant kit (Hilden, Germany), according to the manufacturer’s protocol. The initial yield and purity of RNA were measured using a Nanodrop spectrophotometer 2000c (Thermo Scientific, Waltham, MA, USA) at 260 and 280 nm and agarose gel electrophoresis. An Agilent Bioanalyzer 2100 (Agilent Technologies, USA) was used to analyse the RNA integrity number (RIN). High-quality total RNA (RIN > 7) was provided to Novogene (Beijing, China) for cDNA synthesis (cDNA Rapid Library Preparation Kit, Roche, Mannheim, Germany) and paired-end Illumina HiSeq 2500 sequencing (Illumina, San Diego, CA, USA).

### De novo assembly and functional annotation

Two reference transcriptome libraries were prepared from two individual plant samples of *F. picosperma* and *F. venosa* leaf and root tissue**.** Quality of raw reads of each library were checked separately using FastQC [61] and Trimmomatic [62]. Trimmed reads of the different RNA-seq libraries for *F. picrosperma* and *F. venosa* were merged separately prior to assembly using Trinity [63], which applies a de novo reconstruction method. Quality of the assembly was assessed using the built-in Trinity Perl script to generate an N50 value. Alignment coverage rate was calculated using the program Bowtie [64] with a cut-off set at 70%. Following assembly, Transdecoder was used to predict open reading frames (ORFs) with default parameter of minimum 100 amino acids ORF length. Sequence datasets can be found in the NCBI (NCBI; www.ncbi.nlm.nih.gov), Sequence Read Archive (SRA) database under the accession number PRJNA687112. The assembled protein sequences were used as queries against NR protein database (NCBI non-redundant protein sequences), Nt (NCBI non-redundant nucleotide sequences), Swiss-Prot (a manually annotated and reviewed protein sequence database), KO (KEGG Ortholog database) and GO (Gene Ontology) to identify putative protein functions using the BLAST algorithm with an E-value cut-off of 10^− 5^ [65].

### Classification and characterization of *Fontainea P450* genes

An integrated HMM-search and InterProScan-verification approach was applied to identify the putative *P450* gene families in *Fontainea* species. The P450 family HMM model was used through HMMER3 [66]. The filtered sequences were further blasted using NCBI (https://www.ncbi.nlm.nih.gov/) with the cut-off E-value of 10^− 5^. Sequences annotated as P450 members were collected. The full-length P450 proteins were identified manually using nucleotide sequences in ExPASy [67] based on Chen et al. [30] and after annotation those sequences that did not match with any plant species were discarded. After filtering, the coding sequences of the resultant subjects were retrieved. Finally, results from the two methods were integrated and corrected manually. A BLAST search of *F. picrosperma* and *F. venosa P450* genes against 814 previously identified *P450* genes from 4 different plant species (*A. thaliana, R. communis, M. esculenta, S. lycopersicum*) was performed for *P450* gene classification.

All full-length *P450* genes were named according to the standard P450 nomenclature [21]. Briefly, 40, 55 and 97% sequence identities were used as cut-offs for family, subfamily and allelic variants, respectively. According to a previous study [57], functions of P450 clans were identified. We also calculated their instability index (II) using ExPASy tool (https://web.expasy.org/protparam/). Theoretical iso-electric points (PI) and molecular weight (kDa) were used to assess the physicochemical properties of putative P450s for each full-length P450 protein, as predicted by the ExPASy tool (http://www.expasy.org/tools/). The GRAVY values were calculated using GRAVY calculator (http://www.gravy-calculator.de/). The cellular locations were predicted using the TargetP1.1 server with specificity > 0.95 (http://www.cbs.dtu.dk/ services/TargetP/). Furthermore, P450 motifs were confirmed by Multiple Expectation Maximization for Motif Elicitation (MEME) program with the motif number set to 10 and all other parameters were default (http://alternate.memesuite. org/) [68]. P450 protein sequences were collected from *F. picrosperma, F. venosa* and other species that have appropriate genomic data available: *A. thaliana, R. communis, M. esculenta, S. lycopersicum*. *F. picrosperma.* A pairwise BLASTp comparison was performed using Geneious R11 11.0.2 with default parameters.

### Phylogenetic analysis of P450

One hundred and three (103) full-length genes in *F. picrosperma* and 123 genes in *F. venosa* were used for phylogenetic representation in *Fontainea* species*.* Sequence alignment was performed using Geneious 11.02 software performing ClustalW alignment. The phylogenetic tree was constructed using FastTree and maximum likelihood (ML) algorithm. The statistical bootstrap support of each branch was assessed by re-sampling the amino acid positions 1000 times. The maximum likelihood phylogenetic tree and evolutionary analyses were carried out using iTOL web server (https://itol.embl.de/) [69]. For conserved domain identification, multiple sequence alignment of full-length *Fontainea* species protein sequences were carried out using ClustalX program using default parameters [70]. The alignment file was submitted to Web Logo generator software for generating the logo of conserved domains available at (http://weblogo.berkeley.edu/) [71].

### Relative *P450* gene expression

Total RNA was extracted from leaf and root tissue of 3 different *F. picrosperma* and *F. venosa* plants using an RNAeasy plant extraction kit from QIAGEN® (Hilden, Germany) according to Mitu et al. [12]. The expression levels of *P450* genes were calculated using the CLC genomic 11.01 software package following default parameters. Raw counts for RNA-sequencing data of *F. picrosperma* and *F. venosa* genes were normalized to Transcripts Per Million (TPM). Levels of expression were represented as the log2 ratio of transcript abundance between leaf and root tissues. Next, we generated a z-score for sequencing depth normalized reads counts. Expression of each enzyme in leaf and root tissue was analysed by normal clustering. Relative expression profiles of *P450* genes were presented in the form of a heatmap, which was constructed using z-score with Clustvis (https://biit.cs.ut.ee/clustvis/) [72], using default parameters and a hierarchical clustering analysis to assess biological sample relatedness. We also identified homolog sequences in both *Fontainea* species (percentage of identity 92%). Another heatmap was constructed with z-score of average TPM value of 3 different plant of each species**.** To determine the statistically significant differences between leaf and root tissue of homolog sequences, we used Microsoft Excel software 2013 to conduct Student’s *t*-test. Values are reported as average z-score from three different plant of *Fontainea* species. Significant differences: *p* < 0.05.

### Phylogenetic and quantitative analysis of diterpenoid *P450*

Previously identified diterpenoid *P450*s genes from members of the Euphorbiaceae family were acquired from NCBI (NCBI; www.ncbi.nlm.nih.gov), then used to identify *Fontainea* diterpenoid *P450*s genes, by homology. Identified genes were used to construct a phylogeny tree using Geneious 11.02 software following ClustalW alignment. The phylogenetic tree was constructed using the maximum likelihood method, as described above. For quantitative analysis, RNA-seq from leaf and root tissue from 12 *F. picrosperma* individuals were used. The high-quality cleaned reads were aligned to the *F. picrosperma* reference transcriptomes using CLC genomic workbench 11.01 following default settings. To identify their gene expression values, TPMs value was calculated, as described above and a bar graph was created with their average TPM value. Diterpenoid genes showing statistically significant differential expression between root and leaf tissue of *F. picrosperma* were determined by Student’s *t-*test using Microsoft Excel software 2013. Values were reported as average TPM value from 12 different plant of *F. picrosperma*. Significant differences: *p* < 0.01.

## Supplementary Information

**Additional file 1: Additional Figure 1.** HPLC-UV identification of Tigilanol Tiglate in leaf, root, bark and fruit tissues of *F. picrosperma.* **Additional Figure 2.** Phylogenetic analysis of *P450* genes in *Fontainea* and four other plants species. Total list of genes used in tree can be found in Additional File 1. **Additional Figure 3.** Expression profiles of 103 and 123 full-length *P450* genes in *Fontainea picrosperma* and *Fontainea venosa*, respectively, based on RNA-seq experiment with their CYP ID. Heatmap reflects the relative gene expression in 3 different plants of leaf and root tissue in *F. picrosperma* and *F. venosa*. **Additional Figure 4.** Heatmap showing the genes with significantly differentially expressed based on comparison between leaf and root in *Fontainea picrosperam* and *Fontainea venosa* and significantly different (*p* < 0.05).

**Additional file 2.** Full-length sequences *P450* genes of *Fontainea picrosperma*, *F. venosa* and other plants (*Arabidopsis thaliana, Ricinus communis, Manihot esculenta, Solanum lycopersicum*).

**Additional file 3.** Relative gene expression of full-length *P450* genes of *Fontainea picropserma* and *Fontainea venosa* across 3 different plants (leaf and root) and raw TPM values for diterpenoid biosynthesis genes across 12 different plant (leaf and root) samples and their *P*-value. Yellow color shows the 3 original samples in 3 different plants (leaf and root).

## Data Availability

The raw sequence data from this study have been deposited in the publicly accessible NCBI Sequence Read Archive (SRA) database as accession number PRJNA687112. All data generated and used in this article is included as Additional Figures 1-4 and Additional Files 2 and 3.

## Abbreviations

P450: Cytochrome P450
EF1α: Elongation factor 1-alpha
kDa: kilodalton
GAPC: Glyceraldehyde-3-phosphate dehydrogenase 1, cytosolic
GRAVY: Grand average of hydropathy value
TT: Tigilanol tiglate
TPM: Transcripts Per Million
