# Supplementary material for: The P450 multigene family of Fontainea and insights into diterpenoid synthesis
Source: BMC Plant Biol. 2021 Apr 20;21:191. doi: 10.1186/s12870-021-02958-y (PMC8058993; doi:10.1186/s12870-021-02958-y)
Supplement: Supplementary file 1 — Additional file 1: Additional Figure 1. HPLC-UV identification of Tigilanol Tiglate in leaf, root, bark and fruit tissues of F. picrosperma. Additional Figure 2. Phylogenetic analysis of P450 genes in Fontainea and four other plants species. Total list of genes used in tree can be found in Additional File 1. Additional Figure 3. Expression profiles of 103 and 123 full-length P450 genes in Fontainea picrosperma and Fontainea venosa, respectively, based on RNA-seq experiment with their CYP ID. Heatmap reflects the relative gene expression in 3 different plants of leaf and root tissue in F. picrosperma and F. venosa. Additional Figure 4. Heatmap showing the genes with significantly differentially expressed based on comparison between leaf and root in Fontainea picrosperam and Fontainea venosa and significantly different (p < 0.05). [file 12870_2021_2958_MOESM1_ESM.docx]

**The *P450* multigene family of *Fontainea* and insights into putative diterpenoid synthesis**

Shahida A. Mitu^1,2^, Steven M. Ogbourne^1,2^, Anne H. Klein^1,2^, Trong D. Tran^1^, Paul W. Reddell^3,^ Scott F. Cummins^1,2*^

^1^ GeneCology Research Centre, University of the Sunshine Coast, Maroochydore DC 4558, Queensland, Australia

^2^ School of Science, Technology and Engineering, University of the Sunshine Coast, Maroochydore DC 4558, Queensland, Australia

^3^ EcoBiotics Ltd, Yungaburra, 4884, Queensland, Australia


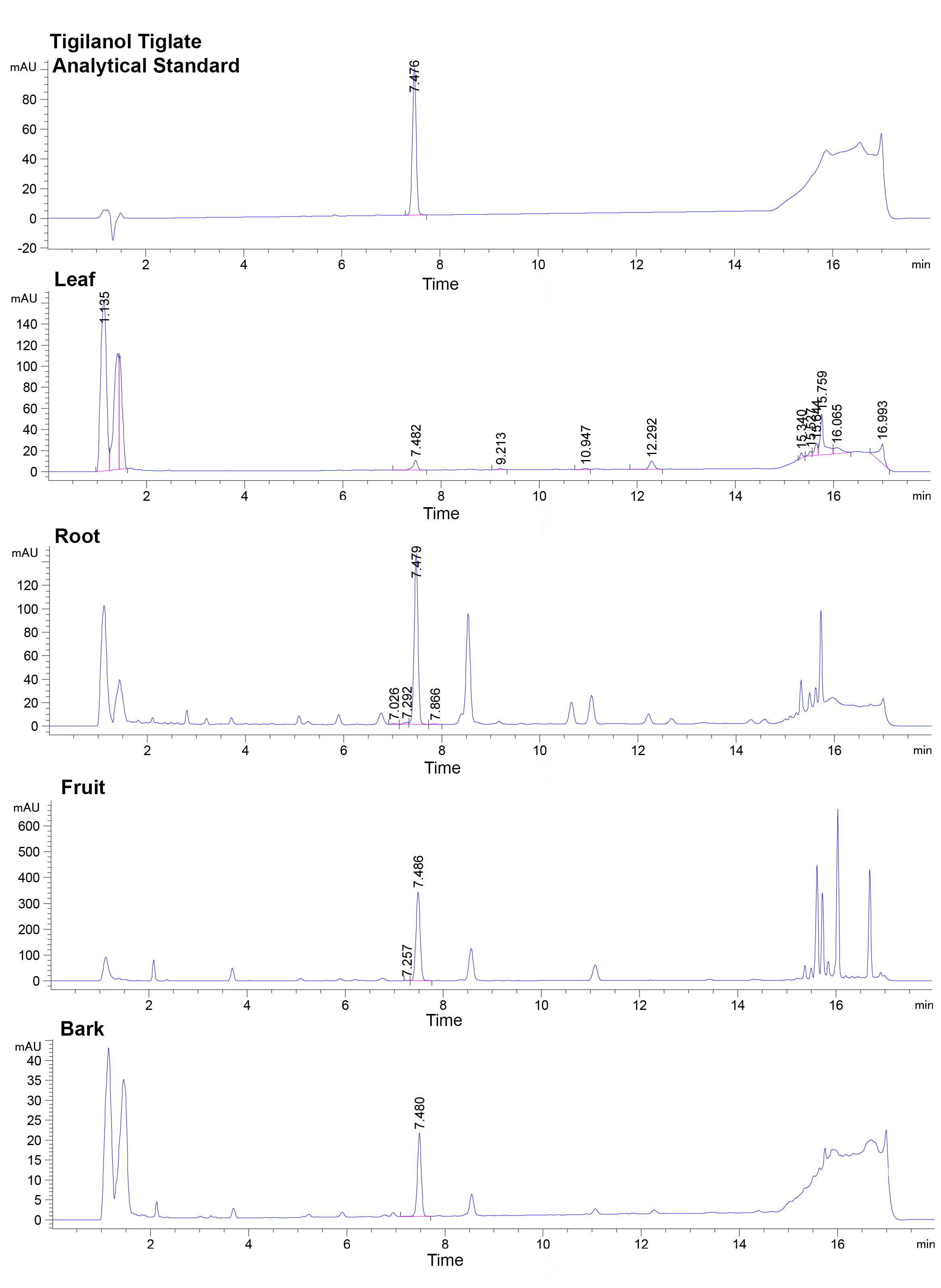


**Additional Figure 1:** HPLC-UV analysis of Tigilanol Tiglate in leaf, root, bark and fruit tissues of *F. picrosperma.* mAU, mass absorbance units.

**
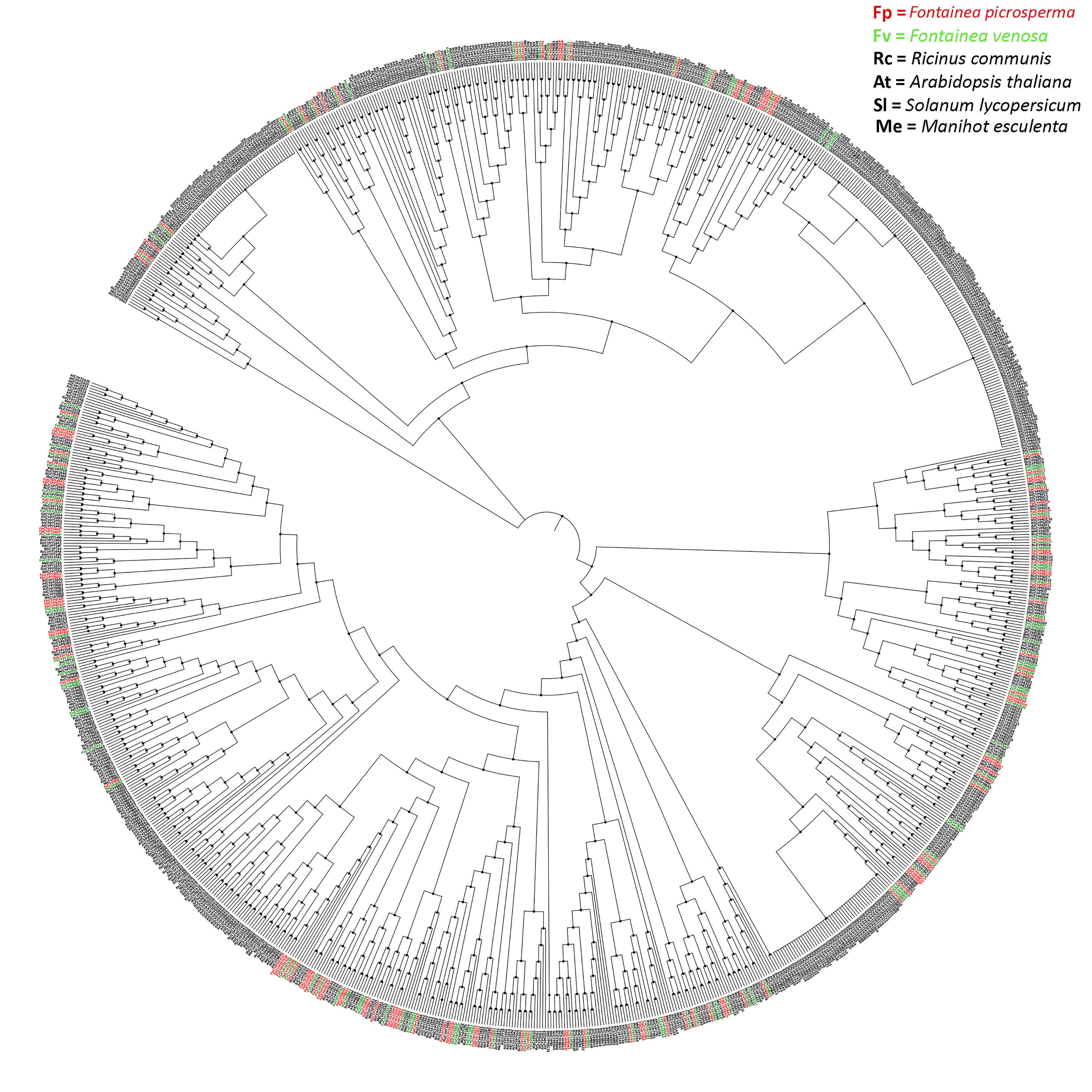
Additional Figure 2**. Phylogenetic analysis of *P450* genes in *Fontainea* and four other plants species.


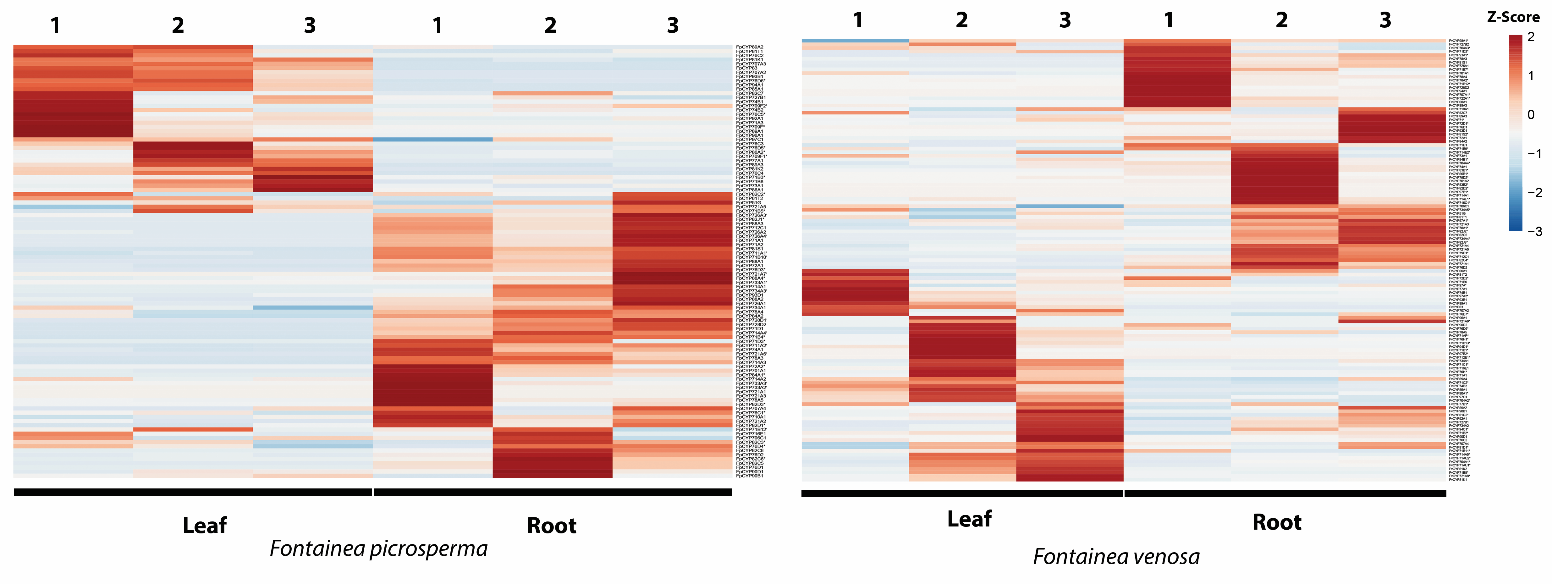


**Additional Figure 3**. Expression profiles of 103 and 123 full-length *P450* genes in *Fontainea* *picrosperma* and *Fontainea venosa*, respectively, based on RNA-seq experiment with their CYP ID. Heatmap reflects the relative gene expression in three different plants of leaf and root separately in *F. picrosperma* and *F. venosa*.

**
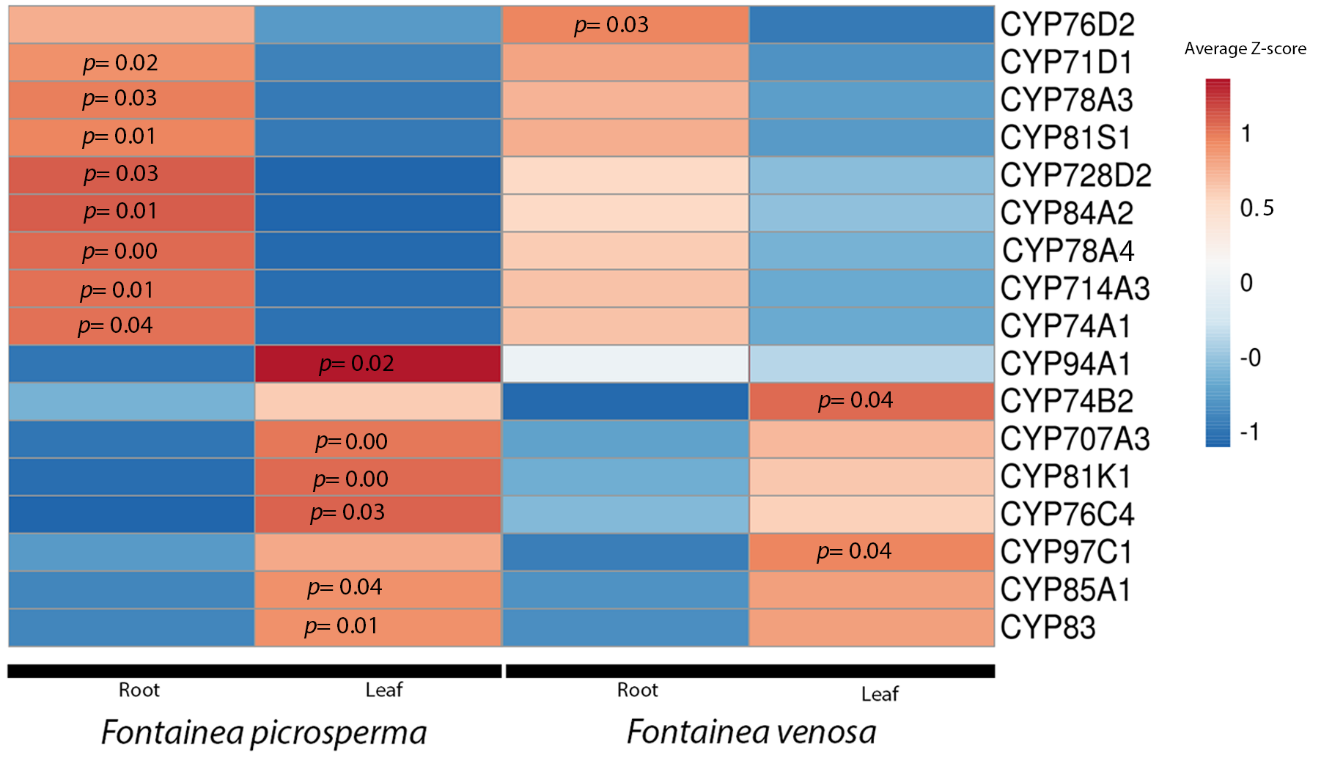
**

**Additional Figure 4**. Heatmap showing the genes with significantly differentially expressed based on comparison between leaf and root in *Fontainea picrosperam* and *Fontainea venosa* and significantly different (*p* < 0.05)
